# Revisiting fatty acid-mediated antibody purification from plasma with insights into selectivity and protein integrity

**DOI:** 10.1371/journal.pone.0352679

**Published:** 2026-07-01

**Authors:** Sirisak Sathorn, Garnpimol C. Ritthidej, Wanatchaporn Arunmanee

**Affiliations:** 1 Queen Saovabha Memorial Institute, Thai Red Cross Society, Bangkok, Thailand; 2 Graduate Program of Industrial Pharmacy, Faculty of Pharmaceutical Sciences, Chulalongkorn University, Bangkok, Thailand; 3 Department of Pharmaceutics and Industrial Pharmacy, Faculty of Pharmaceutical Sciences, Chulalongkorn University, Bangkok, Thailand; 4 Department of Biochemistry and Microbiology, Faculty of Pharmaceutical Sciences, Chulalongkorn University, Bangkok, Thailand; 5 Center of Excellence in Cancer Cell and Molecular Biology, Faculty of Pharmaceutical Sciences, Chulalongkorn University, Bangkok, Thailand; Islamic Azad University, IRAN, ISLAMIC REPUBLIC OF

## Abstract

Therapeutic antibodies play an essential role in modern biopharmaceuticals, with polyclonal antibodies (pAbs) remaining indispensable for applications such as toxin and virus neutralization. However, pAb purification is complicated by serum-derived contaminants. Selective impurity precipitation using caprylic acid (C8) or sodium caprylate (NaC8) provides an effective strategy for obtaining high-purity antibody preparations and serves as a low-cost, scalable non-chromatographic alternative. However, the influence of fatty acid chain length and ionic form on differential precipitation remains poorly understood. Here, we systematically evaluated free fatty acids with varying chain length (C8–C10) and their corresponding sodium salts for pAb purification. Using model proteins, free fatty acids exhibited greater selectivity than their salt forms, and precipitation efficiency decreased with increasing chain length (C8 > C9 > C10). Importantly, C9 at 2% (v/v) provided a favorable balance between impurity removal and γ-globulin retention, achieving effective depletion of albumin while minimizing antibody loss relative to conventional C8 precipitation. Multi-spectroscopic analyses confirmed that γ-globulin maintained its native structure following fatty acid–based precipitation. When applied to the fractionation of IgG from hyperimmunized equine plasma, C9 achieved impurity reduction and IgG homogeneity comparable to conventional C8 treatment while preserving antigen-binding avidity. Collectively, these findings identify C9 as a selective and function-preserving precipitant with potential as an efficient and scalable pretreatment step in polyclonal antibody purification workflows.

## 1. Introduction

Therapeutic antibodies have emerged as a cornerstone in modern biopharmaceuticals due to their remarkable specificity and efficacy in treating a wide range of diseases. Advances in synthetic biology have driven the rapid development and commercialization of monoclonal antibodies (mAbs), leading to a steady growth in new antibody-based therapies and biosimilars [1,2]. However, despite the prominence of mAbs, polyclonal antibodies (pAbs) derived from hyper-immunized animal serum continue to hold an irreplaceable role in medical applications. Their unique ability to target a broad spectrum of epitopes makes them highly effective for the neutralization of toxins and viruses, highlighting their critical importance in managing acute and life-threatening conditions [3]. However, the purification of pAbs from hyperimmunized animal sera remains challenging due to the presence of numerous contaminants.

While pAbs purification protocols are often designed with a final polishing chromatography step to achieve the product purity and quality required for drug substance specifications, the initial non-chromatography separation methods play an equal important role by providing process orthogonality and reducing the impurity burden on subsequent downstream purification operations. Such early-stage fractionation steps contribute not only to product quality but also to improvements in process economics, throughput and overall purification efficiency. Caprylic acid precipitation has been employed for decades as a robust non-chromatographic separation method for the extraction and purification of plasma-derived antibodies and is now widely applied in both laboratory and industrial settings. First introduced by Steinbuch and Audran in the late 1960s, the method utilized neat caprylic acid (C8) as a selective precipitating agent for non-IgG plasma proteins under mildly acid condition [4]. This development was guided by earlier observations that potassium salts of medium chain-length fatty acids (C_6_-C_12_) differed in their capacities to precipitate specific plasma proteins [5]. Subsequent refinements have demonstrated the reliability and scalability of the C8 precipitation technique, particularly its ability to remove broad range of plasma proteins while preserving IgG in solution [6–10]. In contrast to traditional salting-out methods such as sodium and ammonium sulfate precipitation, C8 precipitation eliminates the need for a resolubilization step. Moreover, studies have reported that C8-based fractionation provides higher selectivity and yield while reducing protein aggregation [,^11^,^12^]. These practical advantages have made C8 precipitation a common choice for impurity reduction in many antibody purification workflows. Over the years, the method has become well-established and standardized, primarily in the polyclonal antibody preparations as intact IgG or F(ab′)2 fragment derived from hyperimmune animal plasma, including anti-snake venoms [13,14] and anti-rabies immunoglobulins [15,16].

In plasma-derived products, C8 precipitation effectively removes non-IgG serum proteins, yielding IgG with 90–95% purity [17,18] and approximately 65% retained biological activity in a single step [8]. Although recent studies have reported low aggregation and preserved subclass distribution in C8-fractionated IgG [12], most investigations have focused primarily on overall yield and purity. Earlier work also demonstrated that different purification schemes for plasma-derived antibody fragments can induce structural changes that affect biological potency [19]. Furthermore, the recovery of plasma-derived antibody fragments following C8 precipitation can be varied extensively from 65% to 83.5% [20]. Consequently, structural characterization of intact IgG purified by fatty acid–based fractionation and to optimize product recovery should be explored.

Differential precipitation using C8 and its salt form, sodium caprylate (NaC8), has recently shown promise for downstream processing of CHO-derived mAbs [21–24], with NaC8 offering practical advantages due to its aqueous solubility and ease of preparation as a stock solution. Despite the increasing application of both C8 and NaC8, systematic comparative studies on their selective precipitation behaviors, impurity partitioning profiles, and potential structural impacts remain unexplored. Addressing these gaps is essential for understanding how free fatty acid and its salt form may differentially influence antibody integrity and downstream performance.

Mechanistic insights into the selectivity of protein precipitation induced by fatty acid have been further investigated. Morais and Massaldi [25] proposed that C8-induced precipitation of bovine serum albumin (BSA) at pH value near its isoelectric point (pI of BSA ≈ 4.7–5.1) [26] resulted from direct hydrophobic interactions between C8 molecules and BSA. Such interactions trigger conformational changes and subsequent precipitation cascade of micelle-like macrostructure. In contrast, immunoglobulins, which have relatively basic pI values, mitigate hydrophobicity possibly through charge-induced hydration, allowing them to remain in a dissolved state [21,23]. Hydrophobic interactions therefore play a central role in fatty acid-mediated protein precipitation. The strength of these interaction and adsorption processes is known to be influenced by various factors such as pH, conductivity and temperature [27–29]. A recent study reported that human serum albumin (HSA) exhibits increasing binding affinity for saturated fatty acids as chain length increases from C8 to C14 [30], suggesting that hydrophobic interactions become stronger with longer fatty acid chains. Based on this rationale, the precipitation characteristics of immunoglobins and bovine serum albumin (BSA) induced by various fatty acid-based precipitants with increasing alkyl chain length should be systematically evaluated.

This study aims to evaluate the performance of fatty acid-based precipitants capable of removing major impurities while improving IgG recovery and maintaining its structure and biological activity. Three medium-chain fatty acids (C_8_–C_10_) and their salt forms were revisited as differential precipitants for antibody purification. Precipitation selectivity and efficiency were first assessed using model proteins, followed by multi-spectroscopic structural analysis to evaluate potential conformational effects. These precipitants were then applied to IgG fractionation from hyperimmunized horse plasma at two fixed concentrations, and the resulting fractions were examined for purity profile, homogeneity and antigen-binding performance. By integrating model-protein screening, structural characterization, and application to a complex biological matrix, this work clarifies how fatty acid chain length and chemical form govern precipitation behavior and IgG quality. The findings provide a framework for the rational selection of fatty acid–based precipitants and underscore their potential as economical, high-throughput tools for early impurity reduction in polyclonal antibody manufacturing.

## 2. Materials and methods

### 2.1. Materials and reagents

Caprylic acid (Octanoic acid, ≥ 98%), Pelargonic acid (Nonanoic acid, ≥ 98%), Capric acid (Decanoic acid, ≥ 98%) were obtained from Sigma-Aldrich (USA). Sodium caprylate (Sodium octanoate, > 99%), Sodium pelargonate (Sodium nonanoate, > 99%), Sodium caprate (Sodium decanoate, > 99%) and 8-Anilinonaphthalene-1-sulfonic acid (ANSA) were purchased from TCI (Japan). BSA and γ-globulin were sourced from Merck (Germany). The purchased γ-globulin product consists of the following classes of immunoglobulins: IgG (80%), IgM (10%), and IgA (<10%). Chemicals for sodium dodecyl sulphate-polyacrylamide gel electrophoresis (SDS-PAGE) analysis were obtained from Thermo Fischer Scientific (USA). Standard molecular weight marker and Coomassie blue were from Bio-Rad (USA). Chemicals for the preparation of buffers were of analytical grade and purchased from Ajax Finechem (Australia).

A stock solution of γ-globulin or BSA was freshly prepared at a concentration of 20 mg/ml in normal saline solution (NSS). The solution of water-soluble fatty acid salt was individually prepared as a 10% (w/v) stock solution by dissolving the fatty acid salt in ultrapure water.

### 2.2. Hyperimmunized plasma and snake venom

Hyperimmunized equine plasma against *Naja kaouthia* (NK) venom (lot.no. 23004NK) and freshly reconstituted NK crude venom (1 mg/mL in saline solution) were kindly provided by Queen Saovabha Memorial Institute (Bangkok, Thailand). The plasma was obtained post-collection as part of routine production procedures, and no additional animal experimentation was involved in this study.

### 2.3. Precipitation experiments of model proteins and equine plasma by fatty acid-based precipitants

Small-scale precipitation experiments using model proteins were performed in triplicate in a 1.5-mL microcentrifuge tube. γ-globulin or BSA was diluted with NSS to achieve a final concentration of 10 mg/mL. The pH of the protein solution was then adjusted to 4.9 ± 0.05 using either 1N HCl or 1N NaOH. Following pH adjustment, the fatty acid-based precipitants were slowly added to the mixtures to achieve the final concentration precipitants ranging from 0% to 2% (v/v or w/v). In the case of precipitation experiments with fatty acid salts, the pH was readjusted to 4.9 ± 0.05 to initiate protein precipitation. The precipitation reaction was maintained with stirring (1000 rpm) at 37°C for 45 min in a thermomixer (Eppendorf, Germany). Subsequently, the samples were centrifuged at 5,000 x g for 45 min, and the resulting supernatant was filtered through a 5 µm PTFE syringe filter (Vertical, Thailand). The clarified filtrate from each reaction was then recorded for the net weight and stored at −20 °C for further analysis.

In addition, fatty acid-based precipitation was applied to anti-NK hyperimmunized equine plasma to evaluate the applicability of the selected precipitants under plasma conditions. This procedure followed the same protocol previously described above, using 1% or 2% precipitants, except that net weights were not recorded.

### 2.4. Determination of protein concentration

The total protein concentration of samples was determined in triplicate using the BCA assay (Pierce BCA protein assay kit, USA) following the manufacturer’s protocol in a 96-well plate. Absorbance was measured spectrophotometrically at 562 nm using a microplate reader (CALIOstar, BMG Labtech, Germany). The total protein concentration was then calculated relative to the standard calibrator, either γ-globulin or BSA, corresponding to the sample being analyzed.

### 2.5. Construction of precipitation curve

The protein precipitation data from the small-scale precipitation experiment were calculated for fraction removal based on the mass balance (eq. 1), assuming that the density of the model protein solution is equal to that of water and remains constant.


$$\begin{array}{c} \text{Fraction removal }=\;\frac{{\left[\text{Protein}\right]}_{\text{i}}{\text{Wt}}_{\text{i}}-{\left[\text{Protein}\right]}_{\text{f}}{\text{Wt}}_{\text{f}}}{{\left[\text{Protein}\right]}_{\text{i}}{\text{Wt}}_{\text{i}}} \end{array}$$ (1)

Where [Protein]_i_ and [Protein]_f_ represent the initial and final protein concentrations of sample, respectively, while Wt_i_ and Wt_f_ denote the net weight of the sample before and after treatment with the fatty acid-based precipitant.

The protein precipitation curve was then plotted to represent the fraction of protein that had been removed in respect to precipitant concentration (in %), enabling the study of protein precipitation behavior at a steady increment of precipitant concentration [31,32]. The sample curves were fitted with a sigmoidal function using an asymmetric 5-parameter logistic (5PL) model in GraphPad Prism 10 for data visualization.

### 2.6. Analytical procedures

#### 2.6.1. Sodium dodecyl sulfate–poly acrylamide gel electrophoresis (SDS-PAGE).

Non-reducing, one-dimensional SDS-PAGE was performed to separate proteins by their molecular mass. Protein samples were equally loaded at 2 μL (≤10 μg) on 10% Tris-HCl gel. The loaded gels were subjected to electrophoresis at 100 volts in TGS buffer for 1 h using a Mini Gel tank, (Thermo Fischer Scientific, USA). The separated protein components were stained with Coomassie blue and their molecular weights were estimated by comparison with broad range protein markers (Precision Plus Protein^TM^, Bio-Rad, United Kingdom).

#### 2.6.2. Far-ultraviolet circular dichroism (far-UV CD).

The secondary structure of γ-globulin before and after treatment with fatty acid-based precipitants was determined by far-UV CD with a CD spectropolarimeter (J-815, Jasco, Japan). Each γ-globulin sample was diluted to 400 μg/mL in 20 mM PBS, pH 6.9 prior to analysis and loaded in a 2-mm path length quartz cuvette. The CD spectra of γ-globulin solution were recorded at 20°C from 190 to 280 nm with a wavelength interval of 0.5 nm and a scanning speed of 50 nm/min. The reported spectra of the ellipticity (mdeg) as a function of wavelength were baseline corrected with corresponding buffer diluent.

#### 2.6.3. Intrinsic fluorescence spectroscopy.

Either untreated or treated γ-globulin with various types of fatty acid-based precipitant (0.5% and 1%) was diluted with 20 mM Tris-HCl, pH 7.4 to a concentration of 200 μg/mL. The emission spectra were recorded using a fluorescence spectrophotometer (Cary Eclipse, Agilent, USA) at room temperature. A 1-cm quartz cuvette was used with a fixed excitation wavelength at 295 nm to selectively excite Trp residues [33], and emission spectra were observed between 305–450 nm with both slits set to a width of 5 nm. The emission fluorescence spectra were background-subtracted using a blank.

#### 2.6.4. ANSA probed surface hydrophobicity.

The surface hydrophobicity of the γ-globulin solution was assessed by extrinsic fluorescence spectroscopy using ANSA dye, following a previous study with minor modifications [34]. A 4 mL solution of γ-globulin (50 μg/mL in 20 mM Tris-HCl, pH 7.4) was mixed with 15 μL of ANSA solution (5 mM in DMSO) and the fluorescence intensity of the protein-ANSA complex was measured using a fluorescence spectrophotometer (Cary Eclipse, Agilent, USA) at 475 nm with excitation at 380 nm in a 1-cm quartz cuvette. Background fluorescence was corrected using blank buffer. For the positive control, the stock solution of γ-globulin (20 mg/mL) was diluted in the same manner and boiled at 95°C for 5 min for heat denaturation before testing. The results of independent samples (n = 3) were analyzed by one-way ANOVA followed by Tukey’s multiple comparison test, with *p*-value less than 0.05 considered statistically significant.

#### 2.6.5. Size exclusion chromatography (SEC).

SEC with UV detection at 280 nm was used to monitor the purity of fractionated IgG and the profile of crude plasma. The SEC was performed on a Superdex 200 Increase 10/300 GL column (Cytiva, USA) using 100 mM sodium phosphate buffer containing 150 mM sodium chloride as the running buffer. Samples of crude plasma or fractionated IgG (2.5 mg/mL) were prepared by diluting with running buffer and injected (500 µL) at a flow rate of 0.5 ml/min on an ÄKTA™ pure Fast protein liquid chromatography system (Cytiva, USA).

#### 2.6.6. Avidity Enzyme-Linked Immunosorbent Assay (Avidity ELISA).

A modified indirect ELISA incorporating urea as a chaotropic agent was used to assess the avidity of antigen-specific antibodies in crude plasma and semi-purified IgG samples. Microtiter plates were coated overnight at 2–8 °C with 50 µL/well of NK venom (10 µg/mL in 50 mM carbonate buffer, pH 9.6). After washing with PBS containing 0.05% Tween-20 (PBS-T), wells were blocked with 200 µL of 3% BSA in PBS-T for 30 min at room temperature. Following additional washes, test samples including crude horse anti-NK plasma, fatty acid-fractionated anti-NK IgG, and blank controls were diluted 1:100,000 in 0.3% BSA/PBS-T and added in triplicate (50 µL/well), then incubated at 37 °C for 1 h. To disrupt antigen–antibody interactions, 10 M urea in PBS-T was applied during the wash step. This concentration was selected based on preliminary results on reference IgG preparations showing nearly 50% OD reduction compared to untreated controls (S1 Fig). After washing, 50 µL of HRP-conjugated anti-horse IgG (Sigma-Aldrich, USA) diluted 1:50,000 was added to each well and incubated at 37 °C for 1 h. Plates were then washed and developed with 50 µL of TMB peroxidase substrate (SeraCare Life Sciences, USA) for 30 min in the dark. The enzymatic reaction was stopped by adding 50 µL of 1 N H₂SO₄, and absorbance was measured at 450 nm using a microplate reader (CALIOstar, BMG Labtech, Germany).

Avidity was evaluated by comparing the OD values of test samples treated with urea to those of reference IgG preparations (purified by conventional C8 fractionation under reference condition [18]) without chaotropic treatment. Results were expressed as relative OD (%) using the following formula (eq. 2):


$$\begin{array}{c} \text{Relative OD }(\%)\;=\;\frac{{\text{OD450}}_{\text{sample},\text{ 10M urea}}}{{\text{OD450}}_{\text{reference},\text{ no urea}}}\times \text{100} \end{array}$$ (2)

## 3. Results and discussion

### 3.1. Differential precipitation of model proteins induced by fatty acid-based precipitants

**Fig 1:**
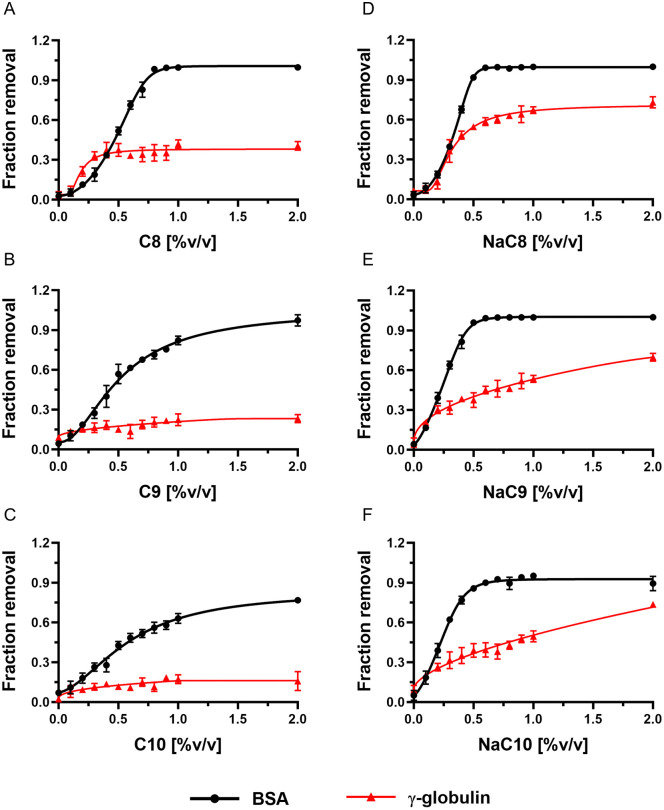
Differential precipitation profiles of BSA and γ-globulin induced by fattyacid–based precipitants. Panels A–C show free fatty acids (C8–C10), and panels D–F show their corresponding sodium salts (NaC8–NaC10). Data are presented as mean ± SD (n = 3), and curves were fitted using a five-parameter logistic model.

The ability of medium chain fatty acids (C8, C9 and C10), along with their water-soluble sodium salts (NaC8, NaC9 and NaC10), to selectively precipitate contaminant proteins was evaluated. With an aim to fractionate polyclonal antibodies derived from animal plasma, BSA was used as the mock contaminant and γ-globulin was represented as the natural source of model antibodies. The precipitation behaviors of both proteins were demonstrated in Fig 1. Across the tested conditions, differential precipitation using fatty acid–based precipitants showed clear distinctions between impurity removal and target protein loss. BSA showed greater susceptibility to precipitation than γ-globulin, and the extent of BSA removal increased with increasing concentrations of these precipitants.

Evidently, fatty acid salts exhibited lower specificity, precipitating a larger proportion of γ-globulin compared to free fatty acids, particularly at higher concentrations. Although all three fatty acid salts (NaC8, NaC9, NaC10) effectively removed BSA, reaching ≥90% removal at approximately 0.6% (w/v), γ-globulin exhibited substantial precipitation under the same concentration, with losses of 39–59% (Fig 1D–F), indicating limited selectivity for the salt forms. This is possible due to protein instability caused by the pH readjustment after adding a stock of fatty acid salts to initiate precipitation by changing the fatty acid salts into their active, unionized soluble forms [24]. Moreover, additional pH adjustments that accompany an increase in salt concentration may also lead to the destabilization of proteins in the solution, which synergistically induces protein precipitation.

Interestingly, precipitation propensities of both BSA and γ-globulin with free fatty acids decreased with longer chain length, following the order C8 > C9 > C10, respectively (Fig 1A-C). While C8 produced near-complete BSA precipitation at 0.8% (v/v), it also caused approximately 35% loss of γ-globulin (Fig 1A). In contrast, γ-globulin showed greater tolerance to C9 and C10, with losses of 22% and 17% at 1% (v/v), compared with 42% for C8, while BSA removal remained high across all three fatty acids (100%, 82% and 63% for C8, C9 and C10, respectively). These results revealed a selectivity window driven by different chain length fatty acids. Although C9 removes impurities slightly less extensively than C8, it offers markedly improved preservation of the target protein. The superior selectivity of C9 became more pronounced at the highest concentration tested (2% v/v). At this level, BSA was precipitated to a similar extent by both C8 and C9, yet γ-globulin loss was lower with C9 (23%) compared with C8 (41%). This more favorable balance between impurity depletion and target protein retention suggests that C9 may serve as a selective impurity-precipitating agent, particularly in early-stage enrichment of antibody-containing fractions.

The reduced precipitation observed with longer-chain free fatty acid can be attributed to their lower solubility [35] and altered interactions with proteins in aqueous system. Although previous studies showed that saturated fatty acids bind to albumin with increasing affinity as chain length increases due to enhanced hydrophobic interactions [30,36], the present results indicate that precipitation behavior depends not only on fatty acid–protein interactions but also on the physicochemical properties of the fatty acids themselves.

In contrast, the influence of chain length on protein precipitation was less evident for fatty acid salts. This deviated from the findings of the initial research conducted by Chanutin and Curnish [5], who reported a gradual depletion of albumin from plasma when using potassium salts of fatty acids, particularly those containing 8–10 carbon atoms. These differences may reflect the distinction between simplified protein systems and complex biological matrices, where interactions among plasma proteins and other components can influence precipitation behavior. However, the differential precipitation observed in pure protein solutions holds potential advantages for identifying fatty acid precipitants that preferentially remove contaminant proteins while exerting minimal impact on antibodies.

### 3.2. Effects of fatty acid-based precipitants on the structure of γ-globulin as model antibodies

**Fig 2:**
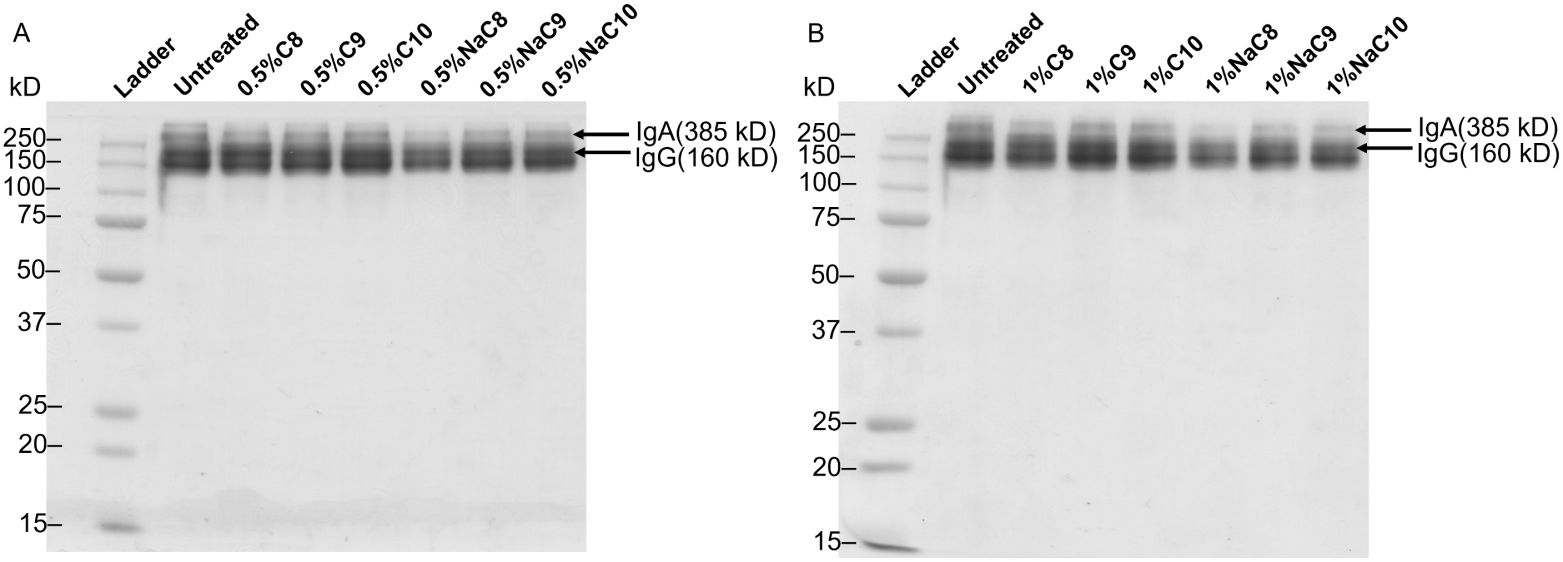
Non-reducing SDS-PAGE analysis of untreated γ-globulin and a set of γ-globulin after treatment with fatty acid-based precipitants, as indicated above each lane. **(A)** Treatment with 0.5% and **(B)** Treatment with 1% precipitants.

Although caprylic acid precipitation has been a long-standing and prevalent method for reducing contaminants in the purification of therapeutic antibodies, it remains important to determine whether variations in the conformation and structural integrity of the protein occur after the fractional precipitation by other types of fatty acid. This investigation involved comprehensive biophysical characterizations aimed at analyzing the biophysical attributes of a model γ-globulin, which serves as a representative for antibody molecules after fractionation with two concentrations (0.5% and 1%) of free fatty acids and their water-soluble salts. These concentrations were selected based on the screening results, showing that most fatty acids reached maximal BSA precipitation at ~1%, while 0.5% enabled assessment of structural effects below the plateau region. Non-reducing SDS-PAGE displayed comparable γ-globulin profiles across all treatments and untreated control (Fig 2). The bands of IgG apparently appeared at 150 kDa across all tested samples with faint bands above 250 kDa protein markers, corresponding to trace IgA (385 kDa) naturally present in γ-globulin products. Importantly, no evidence of protein fragmentation was observed.

Besides assessing the integrity of γ-globulin through SDS-PAGE analysis, considering that the samples were uniformly loaded (2 μL/lane), the lower band intensity observed when treating γ-globulin with 0.5% NaC8 (Fig 2A) and 1% NaC8 (Fig 2B) suggests that a greater proportion of protein was lost during the precipitation process compared to other fatty acid-based precipitation methods. This observation aligns with the results of the precipitation experiment, where NaC8, particularly at 0.5% and 1% concentrations, non-selectively precipitated a significant portion of γ-globulin, resulting in the removal of 55% and 67%, respectively (Fig 1D).

**Fig 3:**
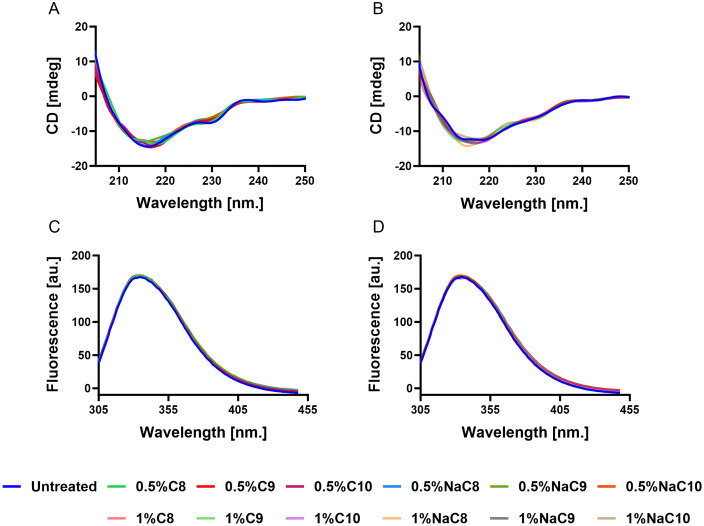
Spectroscopic analysis of γ-globulin treated with fatty acid–based precipitants. Far-UV circular dichroism spectra of γ-globulin treated with (A) 0.5% and (B) 1% precipitants. Intrinsic fluorescence spectra of γ-globulin treated with (C) 0.5% and (D) 1% precipitants.

Moreover, we conducted evaluations of secondary structure via far-UV CD and tertiary structure through intrinsic fluorescence spectroscopy. These multi-spectroscopic evaluations aimed to investigate whether the presence of fatty acid-based precipitants influenced the conformation changes of γ-globulin. To achieve this, we compared the resulting spectra from γ-globulin samples treated with 0.5% and 1% concentrations of various types of fatty acid-based precipitants to those of the untreated γ-globulin solution. As a result, a positive peak around 195 nm and a corresponding negative peak at approximately 218 nm were consistently observed in all far-UV CD spectra. These findings collectively suggest the prevalence of β-pleated sheets as the predominant secondary structure of all examined samples [37]. The overlay spectra of γ-globulin subjected to treatment with 0.5% (Fig 3A) and 1% (Fig 3B) fatty acid-based precipitants aligned closely with the spectrum of untreated γ-globulin solution. This alignment indicates that the secondary structure of γ-globulin remained well preserved under the influence of these precipitants.

High-sensitivity intrinsic fluorescence spectroscopy serves as a robust tool for detecting alterations in protein folding or variations in the polar microenvironment of aromatic amino acid residues [,^38^]. As illustrated in Fig 3C and 3D, the emission spectra of the γ-globulin displayed a high degree of superimposition, implying the absence of noticeable conformational changes in samples treated with fatty acid-based precipitants compared to the untreated sample. Additionally, this analysis excludes any significant changes in the polarity of the tryptophan (Trp) residue within soluble γ-globulin following fractionation with both 0.5% (Fig 3C) and 1% (Fig 3D) fatty acid-based precipitants.

Taken together, the multi-spectroscopic analyses indicate that soluble γ-globulin retains its secondary and tertiary structural integrity following fractionation with the various fatty acid–based precipitants, including both free acids and their salt forms. As structural assessments in purification workflows are often conducted only after multiple downstream steps [19,39], making it difficult to attribute any structural changes to a specific operation, these results clarify the effect of the fractionation step itself and support the applicability of fatty acid–based precipitation in processes requiring preservation of antibody structural quality.

### 3.3. Fatty acid-based precipitants did not alter surface hydrophobicity of γ-globulin

**Fig 4:**
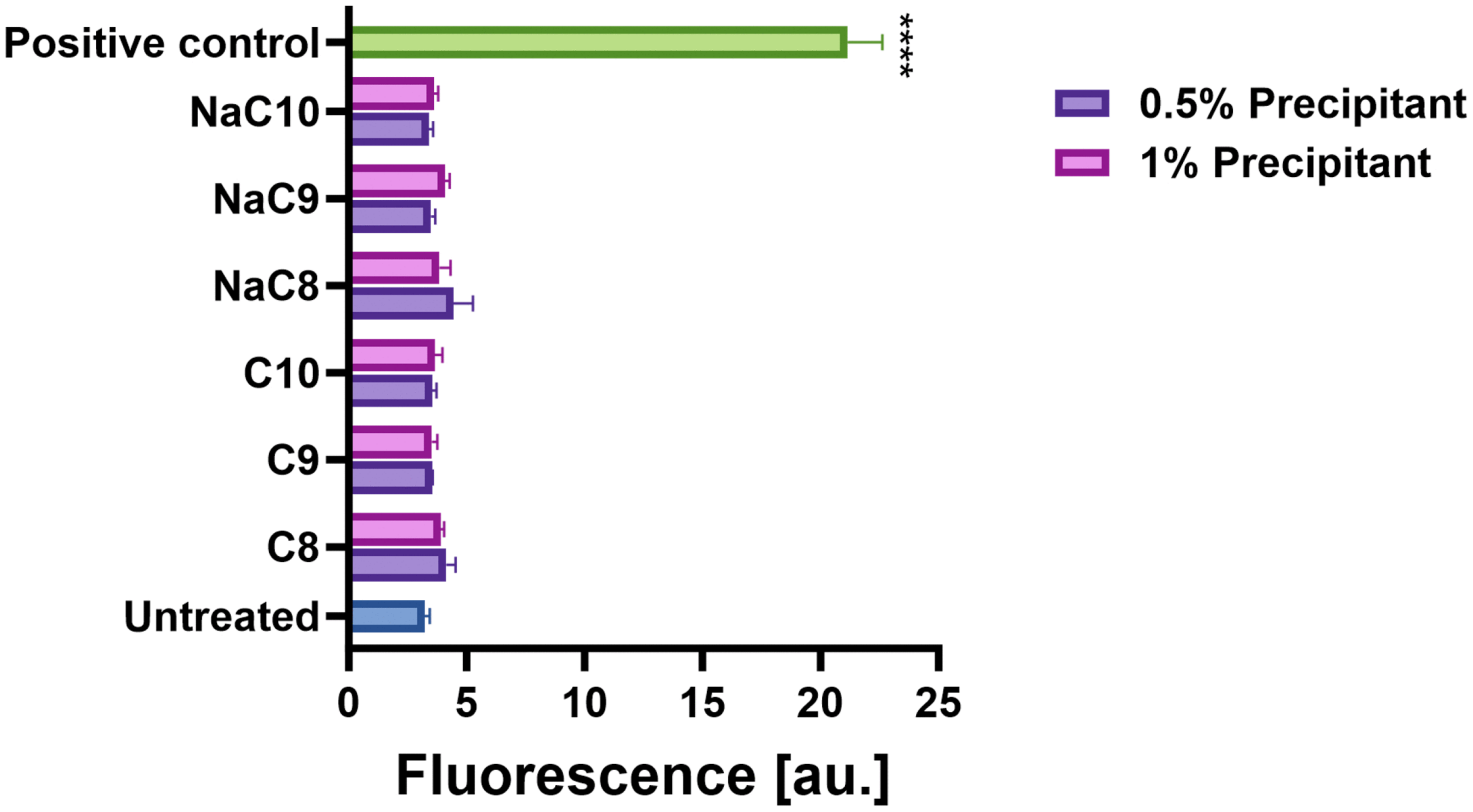
Surface hydrophobicity of untreated γ-globulin and γ-globulin treated with fatty acid–based precipitants (0.5% and 1%). Data represent mean ± SD (n = 3). ****p < 0.0001.

The hydrophobicity of a protein's surface plays a critical role in determining its physical stability, solubility, propensity for aggregation, and adsorption characteristics. Utilizing hydrophobic fluorescent dyes provides a spectroscopic method for assessing the surface hydrophobicity of both native and denatured proteins [34,40]. These dyes interact non-covalently with exposed hydrophobic regions of proteins, producing fluorescence signals that correlate with surface hydrophobicity. In this study, we used ANSA, a hydrophobic fluorescent dye, to assess whether the use of fatty acid-based precipitants at concentrations of 0.5% and 1% impacted the surface hydrophobicity of γ-globulin fractions. Untreated γ-globulin served as the control, while heat-denatured γ-globulin was used as a positive reference to represent a high-hydrophobicity state. The fluorescence intensities of the protein-dye complexes are shown in Fig 4. No significant changes were observed between untreated γ-globulin and γ-globulin treated with either 0.5% or 1% fatty acid-based precipitants. In contrast, heat-denatured γ-globulin exhibited a marked increase in fluorescence intensity, indicating a substantial exposure of hydrophobic regions (*p* < 0.0001). These findings supported that the treatment of γ-globulin with 0.5% or 1% fatty acid-based precipitants during protein fractional precipitation process did not induce alterations in the protein's surface hydrophobicity, as assessed through surface hydrophobicity measurements.

### 3.4. Characteristics of equine IgG fractions obtained from fatty acid-based precipitations

To evaluate the efficiency of fatty acid-based precipitation as an initial purification step for hyper-immunized equine plasma, plasma samples were treated with various fatty acid-based precipitants at concentrations to 1% and 2%. These higher concentrations than screening experiment were chosen to account for the greater protein content of plasma compared with the model systems. Subsequent analysis of the semi-purified fractions showed that the extent of plasma protein precipitation increased with the concentration of fatty acid-based precipitants. Moreover, the total protein content of the fractions obtained with longer-chain fatty acids tended to increase (Table 1), consistent with the reduced precipitation efficiency observed for model proteins as chain length increased.

**Table 1:** Total protein content of crude and fatty acid–fractionated hyperimmunized equine plasma.

Sample/Concentration of FA	FA-based precipitant	[Total protein]^a^; mg/mL
Crude plasma^b^	–	35.55 ± 0.53
1%	C8	15.94 ± 0.69
	C9	17.71 ± 1.02
	C10	21.97 ± 0.18
	NaC8	17.71 ± 0.28
	NaC9	14.51 ± 1.36
	NaC10	15.19 ± 0.47
2%	C8	12.84 ± 0.46
	C9	16.04 ± 0.95
	C10	17.83 ± 1.04
	NaC8	9.45 ± 1.55
	NaC9	10.21 ± 0.76
	NaC10	11.07 ± 0.58

^a^ Data represent mean ± SE of three independent measurements.

^b^ Crude plasma was diluted equal volume with NSS

**Fig 5:**
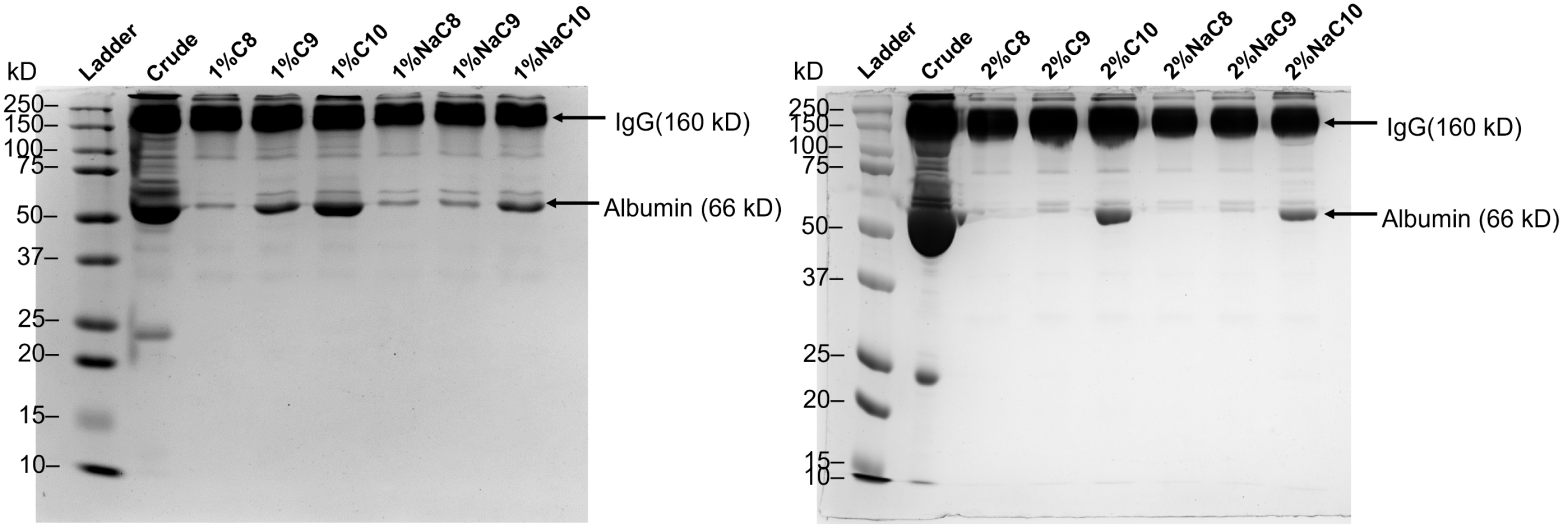
Non-reducing SDS-PAGE analysis of equine crude plasma and plasma fractions obtained using fatty acid–based precipitants at (A) 1% and (B) 2%.

SDS-PAGE analysis of crude and fractionated plasma samples revealed that increasing fatty acid concentration generally improved purity profiles (Fig 5). Distinct impurity patterns were also observed depending on the type of fatty acid used at a fixed concentration. These findings highlighted differences in their capability to remove non-IgG plasma proteins. Notably, fractionation with free fatty acids of increasing chain length (C8 to C10) tends to diminish removal of contaminating proteins, as evidenced by stronger residual bands of non-IgG species. This finding was consistent with precipitation studies involving BSA as a model contaminant. At 2%, most fatty acid-based precipitants effectively provided enriched IgG fractions. However, fractionation with both C10 and NaC10 at 2% failed to completely remove albumin protein, as evidenced by noticeable albumin bands in SDS-PAGE result (Fig 5B). Interestingly, fractionation with 2% C8 and 2% C9 produced semi-purified fractions with comparable purity profiles.

**Fig 6:**
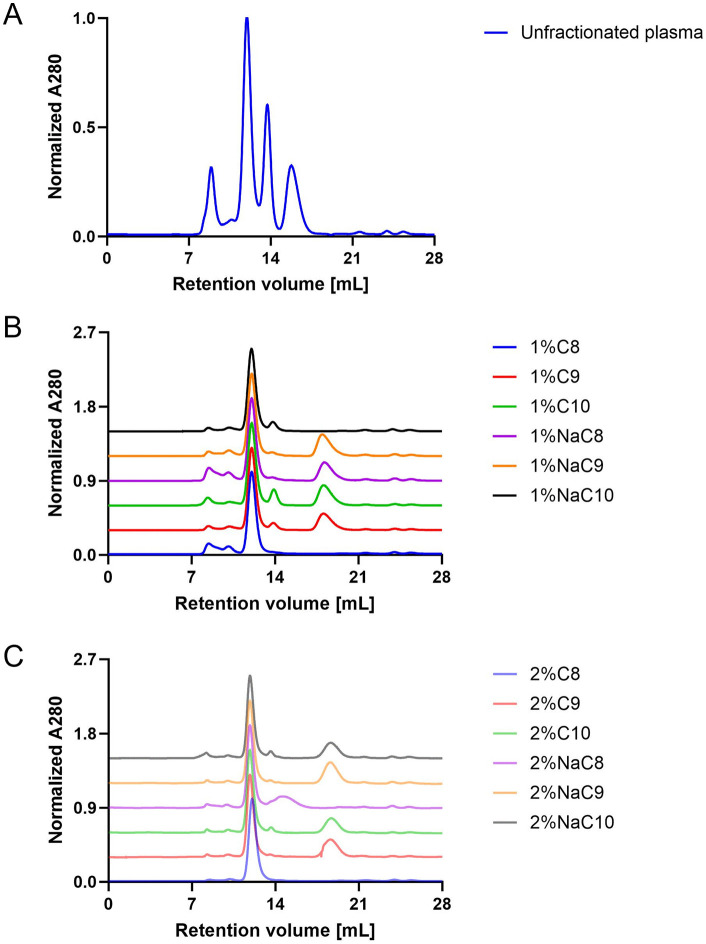
Size-exclusion chromatography analysis of equine plasma fractions. Chromatograms represent (A) equine crude plasma and plasma fractions obtained using fatty acid-based precipitants at concentration of (B) 1% and (C) 2%.

In addition, SEC was used to further assess the homogeneity of fractionated plasma. As shown in Fig 6, the chromatograms of crude plasma (Fig 6A) displayed multiple protein peaks, whereas fractionated samples exhibited a marked reduction in non-IgG components (Fig 6B and 6C), consistent with SDS-PAGE results. In addition to the major IgG peak, broad signals associated with high-molecular-weight species were detected following precipitation with 1% fatty acids, but these signals decreased substantially when the concentration increased to 2%. This emphasized the concentration-dependent efficacy of fatty acid-based precipitation and further optimization required to maximize contaminant removal. The SEC chromatograms also revealed low-molecular-weight species that formed after fractionation with peak eluting at 18 mL in most conditions (Fig 6B and 6C), except for samples treated with 1% C8, 1% NaC10, 2%C8 and 2% NaC8. Although further downstream processing such as ultrafiltration and chromatography can effectively remove such low molecular weight aggregates [18,19], the present results demonstrated the effectiveness of fatty acid-based precipitation in eliminating the majority of non-IgG proteins at an early stage. Regardless of low molecular weight species and the IgG yield, SDS-PAGE and SEC analyses indicated that fractionation with 2% C8- and 2% C9-based precipitants (in free acid and salt forms) produced semi-purified fractions with comparable purity profiles.

### 3.5. Effects on binding quality of fatty acid-based fractionated equine IgG

Preserving antigen-binding properties is an important consideration during antibody purification, as purification strategies may influence antibody composition, structural flexibility, or epitope accessibility, thereby affecting functional binding [41,42]. To evaluate whether fatty acid–based precipitation affects the functional binding quality of anti-NK IgG, the relative avidity of fractionated IgG preparations was assessed and compared with that obtained using a reference C8-based fractionation protocol (2% C8, 23 °C).

**Fig 7:**
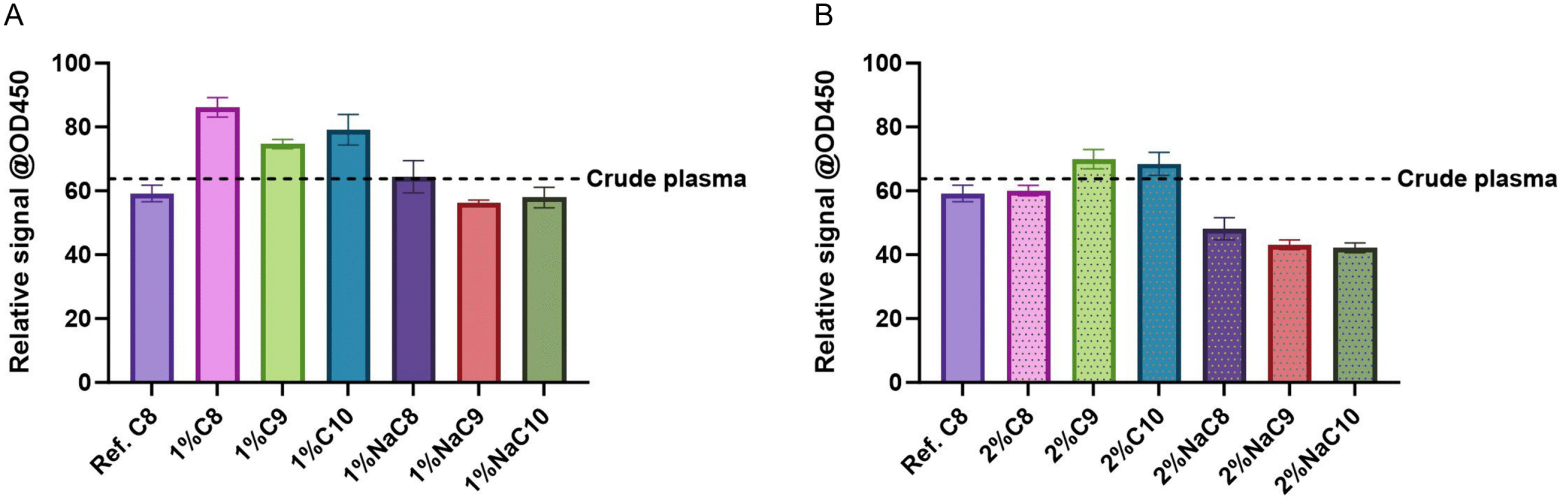
Avidity assessment of horse anti-*Naja kaouthia* (NK) IgG preparations following fatty acid-based fractionation. Semi-purified IgGs obtained with 1% (A) and 2% (B) fatty acid-based precipitants are shown. Results are expressed as relative OD (%) compared with a reference IgG preparation. Data represent mean ± SE of three technical replicates from a single plasma fractionation experiment. The dashed line indicates the relative avidity of crude anti-NK plasma.

As shown in Fig 7, most fatty acid–based precipitants preserved avidity comparable to crude plasma and to the reference C8 fractionation protocol. A noticeable reduction in avidity was observed only with 2% fatty acid salts (Fig 7B), which corresponded with their lower protein recovery (Table 1), suggesting a greater loss of antibodies under these conditions. In contrast, precipitation with 1% free fatty acids produced a modest increase in avidity (Fig 7A), likely reflecting improved removal of non-specific plasma proteins while minimizing loss of target antibodies. Importantly, at 2% (v/v), IgG fractionated with C9 and C10 free fatty acids exhibited relative avidity values slightly higher than crude plasma and C8-based fractionations. This trend is consistent with their higher total protein recovery (Table 1).

Although the definitive confirmation of biological activity, particularly for therapeutic pAbs, requires endpoint *in vivo* assays, avidity measured by ELISA provides a valuable *in vitro* indicator of whether antigen-binding properties are preserved during purification [43]. In this study, the differences observed across conditions are unlikely to indicate substantial alterations in biological function [46]. These results indicate that fatty acid–based precipitation largely preserves the antigen-binding properties of polyclonal antibodies, consistent with previous reports that C8 precipitation maintains high specific activity of plasma-derived antibodies [8,20,44]. The present findings further suggest that C9-mediated precipitation achieves a favorable balance between impurity removal and preservation of functional antibody, supporting its potential application as a selective pretreatment step in plasma-derived antibody purification.

From a practical perspective, C9 has been used in agricultural and food-processing applications [45,46]. Toxicological evaluations indicate low mammalian toxicity, and C9 is also considered Generally Recognized as Safe (GRAS), with regulatory approval for use as a food additive [47,48]. Overall, these results provide a rationale for adopting C9 as an alternative, function-preserving precipitant in early-stage polyclonal antibody purification and offer a foundation for optimizing scalable, cost-effective fractionation strategies that enhance downstream purification efficiency while minimizing antibody loss.

## 4. Conclusions

This study demonstrates that medium-chain fatty acids differ markedly in their ability to drive selective protein precipitation, with both chain length and ionic form governing the balance between impurity removal and antibody retention. Across all conditions tested, free fatty acids provided more favorable selectivity than their sodium salts, and the contrasting precipitation responses of BSA and γ-globulin defined a clear operational window for impurity depletion while preserving antibody recovery. Within this window, C9 emerged as a practical alternative to the conventional use of C8, offering improved discrimination between contaminant and target proteins.

Importantly, fatty acid-based fractionation did not compromise antibody quality. The preservation of native γ-globulin structure and antigen-binding avidity indicates that the physicochemical perturbations introduced by these precipitants are minimal under the tested conditions. Furthermore, the correspondence between model-protein behavior and plasma fractionation performance further supports the utility of chain-length–guided selection of fatty acid precipitants for antibody purification.

## Supporting information

S1 Fig Urea-induced dissociation of anti-NK IgG bound to NK venom in modified ELISA. (DOCX)

S2 Fig Original images underlying all gel results. (PDF)

S3 File Supplementary tables showing minimal dataset. (DOCX)
