# Supplementary material for: Revisiting fatty acid-mediated antibody purification from plasma with insights into selectivity and protein integrity
Source: PLoS One. 2026 Jul 1;21(7):e0352679. doi: 10.1371/journal.pone.0352679 (PMC13322513; doi:10.1371/journal.pone.0352679)

**S2 Fig. Urea-induced dissociation of anti-NK IgG bound to NK venom in modified ELISA.**

Relative avidity (mean ± SD, n = 3) of reference anti-NK IgG preparations determined by modified indirect ELISA in the presence of increasing urea concentrations as a chaotropic agent.


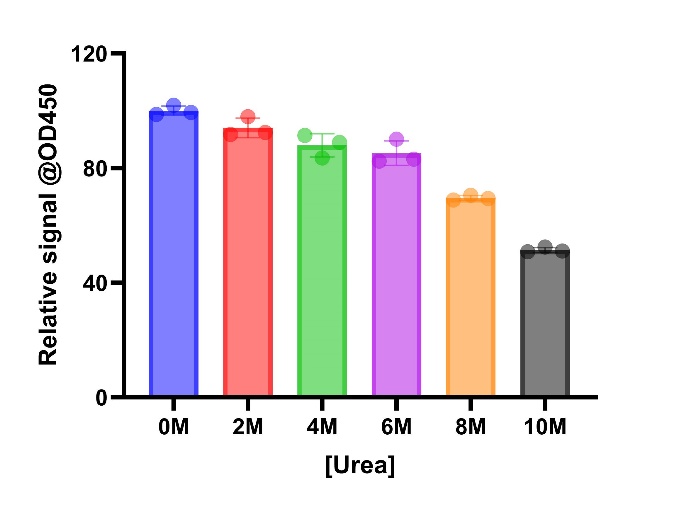

Supplement: S1 Fig — (DOCX) [file pone.0352679.s001.docx]
