# Supplementary figures and images for: Revisiting fatty acid-mediated antibody purification from plasma with insights into selectivity and protein integrity
Source: PLoS One. 2026 Jul 1;21(7):e0352679. doi: 10.1371/journal.pone.0352679 (PMC13322513; doi:10.1371/journal.pone.0352679)

**Original images supported all gel results**

Figure 2A

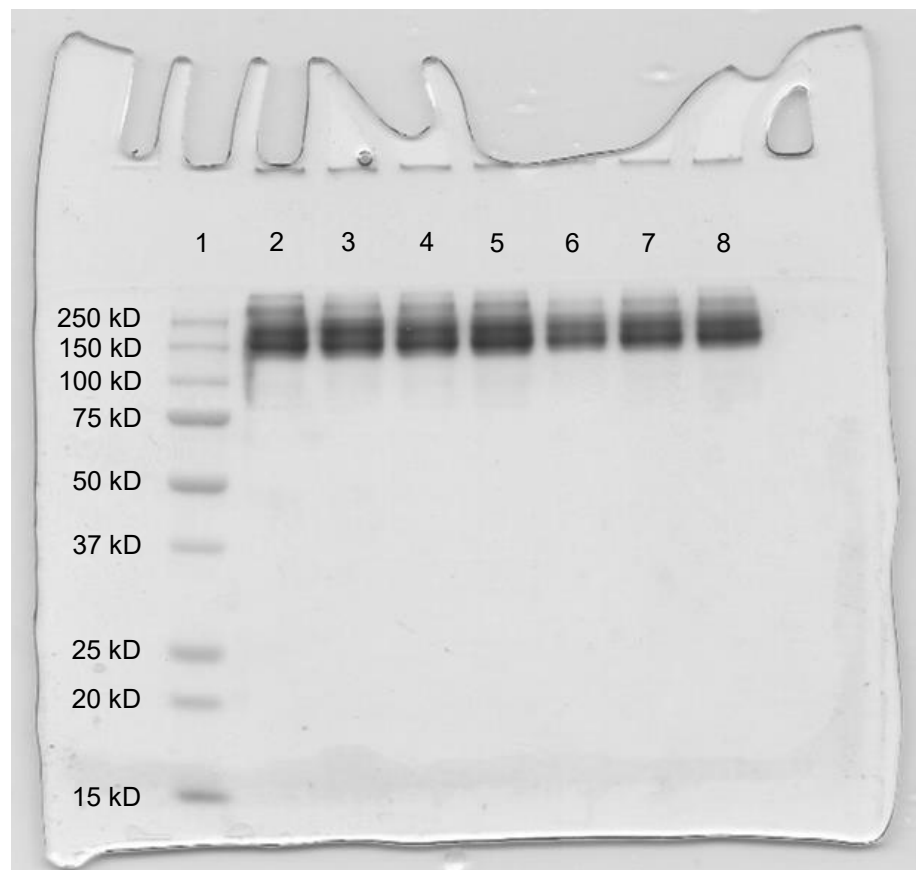

Figure 2B

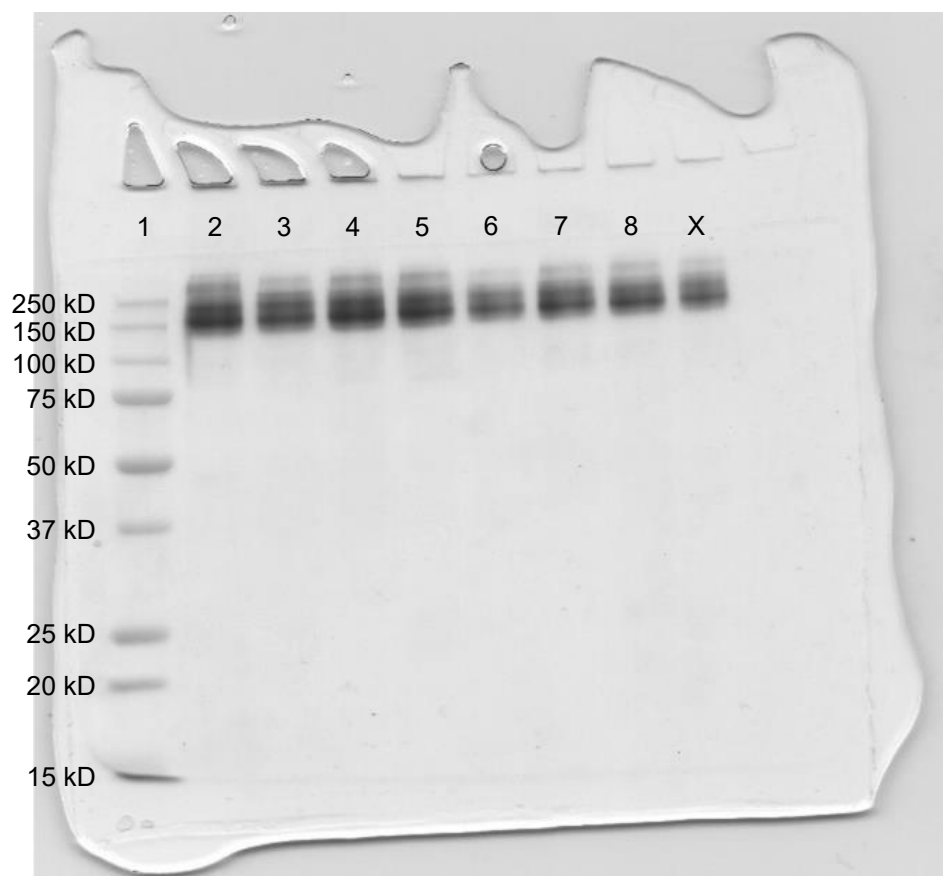

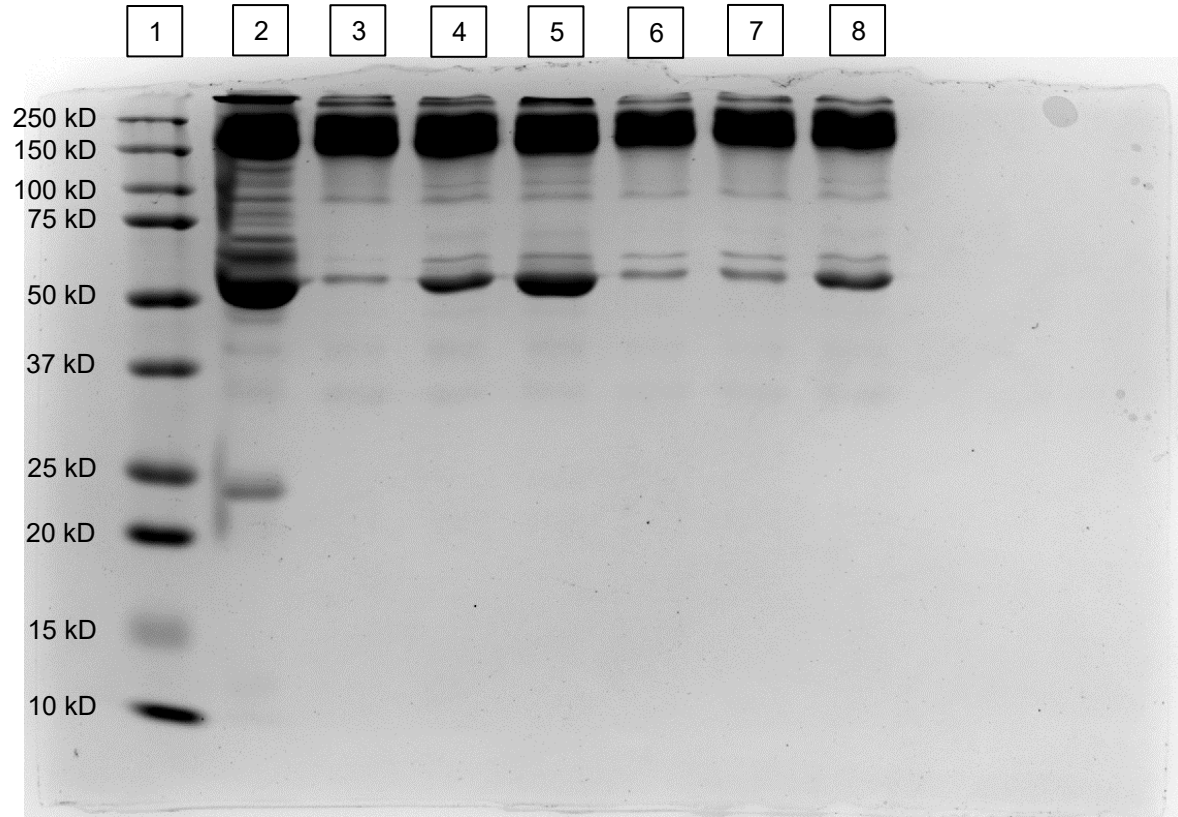

Figure 5B

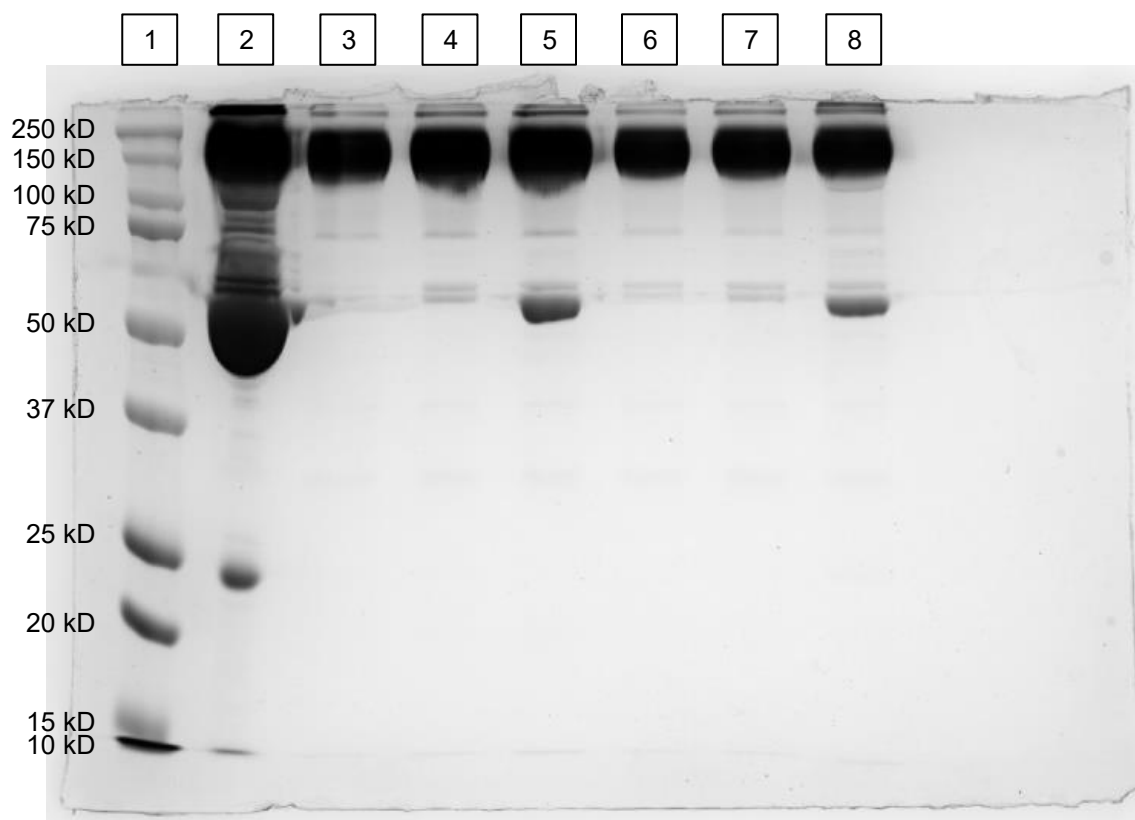

Supplement: S2 Fig — (PDF) [file pone.0352679.s002.pdf]
