# Supplementary material for: Revisiting fatty acid-mediated antibody purification from plasma with insights into selectivity and protein integrity
Source: PLoS One. 2026 Jul 1;21(7):e0352679. doi: 10.1371/journal.pone.0352679 (PMC13322513; doi:10.1371/journal.pone.0352679)
Supplement: S3 File — (DOCX) [file pone.0352679.s003.docx]

**S2. Data availability – Minimal data set**

Fig. 1A

| **C8 [%v/v]** | **BSA fraction removal** | | | | | **γ-globulin fraction removal** | | | | |
| --- | --- | --- | --- | --- | --- | --- | --- | --- | --- | --- |
|  | **n1** | **n2** | **n3** | **Mean** | **SD** | **n1** | **n2** | **n3** | **Mean** | **SD** |
| 0.0 | 0.0360 | 0.0467 | -0.0046 | 0.0260 | 0.0270 | 0.0625 | 0.0100 | 0.0248 | 0.0324 | 0.0271 |
| 0.1 | 0.0247 | 0.0786 | 0.0573 | 0.0535 | 0.0272 | 0.1064 | 0.0478 | 0.0391 | 0.0645 | 0.0366 |
| 0.2 | 0.1182 | 0.1168 | 0.1054 | 0.1135 | 0.0070 | 0.2358 | 0.1705 | 0.2313 | 0.2125 | 0.0365 |
| 0.3 | 0.2296 | 0.1322 | 0.2033 | 0.1884 | 0.0504 | 0.3631 | 0.3024 | 0.3223 | 0.3293 | 0.0309 |
| 0.4 | 0.3421 | 0.3186 | 0.3422 | 0.3343 | 0.0136 | 0.3353 | 0.3441 | 0.4401 | 0.3732 | 0.0582 |
| 0.5 | 0.5448 | 0.4867 | 0.5202 | 0.5173 | 0.0291 | 0.3679 | 0.3291 | 0.4252 | 0.3741 | 0.0484 |
| 0.6 | 0.6793 | 0.7398 | 0.7189 | 0.7126 | 0.0307 | 0.3360 | 0.3300 | 0.3278 | 0.3312 | 0.0042 |
| 0.7 | 0.8631 | 0.8601 | 0.7627 | 0.8286 | 0.0571 | 0.3258 | 0.2944 | 0.3997 | 0.3400 | 0.0540 |
| 0.8 | 0.9935 | 0.9663 | 0.9900 | 0.9833 | 0.0148 | 0.3326 | 0.3018 | 0.4224 | 0.3522 | 0.0626 |
| 0.9 | 0.9965 | 0.9878 | 0.9947 | 0.9930 | 0.0046 | 0.3215 | 0.3169 | 0.4151 | 0.3512 | 0.0554 |
| 1.0 | 0.9961 | 0.9952 | 0.9961 | 0.9958 | 0.0005 | 0.4225 | 0.3873 | 0.4499 | 0.4199 | 0.0314 |
| 2.0 | 0.9971 | 0.9965 | 0.9967 | 0.9968 | 0.0003 | 0.4143 | 0.3702 | 0.4316 | 0.4054 | 0.0317 |

Fig. 1B

| **C9 [%v/v]** | **BSA fraction removal** | | | | | **γ-globulin fraction removal** | | | | |
| --- | --- | --- | --- | --- | --- | --- | --- | --- | --- | --- |
|  | **n1** | **n2** | **n3** | **Mean** | **SD** | **n1** | **n2** | **n3** | **Mean** | **SD** |
| 0.0 | 0.0562 | 0.0371 | 0.0410 | 0.0448 | 0.0101 | 0.0982 | 0.1025 | 0.0680 | 0.0896 | 0.0188 |
| 0.1 | 0.1413 | 0.1041 | 0.0655 | 0.1036 | 0.0379 | 0.1492 | 0.1187 | 0.1410 | 0.1363 | 0.0158 |
| 0.2 | 0.1913 | 0.1791 | 0.1895 | 0.1866 | 0.0066 | 0.1906 | 0.1516 | 0.1355 | 0.1592 | 0.0283 |
| 0.3 | 0.3098 | 0.2303 | 0.2812 | 0.2738 | 0.0403 | 0.1641 | 0.1999 | 0.1274 | 0.1638 | 0.0363 |
| 0.4 | 0.4044 | 0.3155 | 0.4785 | 0.3995 | 0.0816 | 0.2182 | 0.1805 | 0.1520 | 0.1836 | 0.0332 |
| 0.5 | 0.5066 | 0.5486 | 0.6493 | 0.5682 | 0.0733 | 0.1708 | 0.1471 | 0.1337 | 0.1505 | 0.0188 |
| 0.6 | 0.6282 | 0.5932 | 0.6227 | 0.6147 | 0.0188 | 0.1910 | 0.1308 | 0.0835 | 0.1351 | 0.0539 |
| 0.7 | 0.6740 | 0.6701 | 0.6847 | 0.6763 | 0.0076 | 0.2211 | 0.1835 | 0.1572 | 0.1873 | 0.0321 |
| 0.8 | 0.7445 | 0.6809 | 0.7195 | 0.7150 | 0.0320 | 0.2428 | 0.2064 | 0.1687 | 0.2060 | 0.0371 |
| 0.9 | 0.7430 | 0.7669 | 0.7532 | 0.7544 | 0.0120 | 0.2434 | 0.2069 | 0.1997 | 0.2166 | 0.0234 |
| 1.0 | 0.7968 | 0.8085 | 0.8579 | 0.8211 | 0.0324 | 0.2647 | 0.2293 | 0.1764 | 0.2235 | 0.0444 |
| 2.0 | 0.9979 | 0.9955 | 0.9245 | 0.9726 | 0.0417 | 0.2639 | 0.2285 | 0.2043 | 0.2322 | 0.0300 |

Fig. 1C

| **C10 [%v/v]** | **BSA fraction removal** | | | | | **γ-globulin fraction removal** | | | | |
| --- | --- | --- | --- | --- | --- | --- | --- | --- | --- | --- |
|  | **n1** | **n2** | **n3** | **Mean** | **SD** | **n1** | **n2** | **n3** | **Mean** | **SD** |
| 0.0 | 0.0667 | 0.0704 | 0.0759 | 0.0710 | 0.0046 | 0.0230 | 0.0390 | 0.0065 | 0.0228 | 0.0163 |
| 0.1 | 0.1639 | 0.0946 | 0.0736 | 0.1107 | 0.0473 | 0.1307 | 0.0584 | 0.0492 | 0.0794 | 0.0446 |
| 0.2 | 0.1769 | 0.1447 | 0.2207 | 0.1808 | 0.0381 | 0.1035 | 0.0979 | 0.0801 | 0.0938 | 0.0122 |
| 0.3 | 0.2617 | 0.2340 | 0.2957 | 0.2638 | 0.0309 | 0.1311 | 0.0852 | 0.1288 | 0.1150 | 0.0259 |
| 0.4 | 0.2416 | 0.3366 | 0.2563 | 0.2782 | 0.0511 | 0.1462 | 0.1201 | 0.1400 | 0.1354 | 0.0136 |
| 0.5 | 0.3911 | 0.4380 | 0.4489 | 0.4260 | 0.0307 | 0.1305 | 0.1029 | 0.1169 | 0.1168 | 0.0138 |
| 0.6 | 0.4572 | 0.4744 | 0.5209 | 0.4842 | 0.0330 | 0.1317 | 0.0958 | 0.0988 | 0.1088 | 0.0199 |
| 0.7 | 0.5242 | 0.5420 | 0.4822 | 0.5161 | 0.0307 | 0.1859 | 0.1290 | 0.1373 | 0.1507 | 0.0307 |
| 0.8 | 0.5602 | 0.6024 | 0.5195 | 0.5607 | 0.0415 | 0.1463 | 0.0782 | 0.1182 | 0.1142 | 0.0342 |
| 0.9 | 0.5510 | 0.6144 | 0.5733 | 0.5796 | 0.0322 | 0.1943 | 0.1956 | 0.1622 | 0.1840 | 0.0189 |
| 1.0 | 0.6315 | 0.6664 | 0.5925 | 0.6301 | 0.0370 | 0.2030 | 0.1778 | 0.1370 | 0.1726 | 0.0333 |
| 2.0 | 0.7715 | 0.7610 | 0.7701 | 0.7675 | 0.0057 | 0.2381 | 0.1395 | 0.0986 | 0.1587 | 0.0717 |

Fig. 1D

| **NaC8**  **[%w/v]** | **BSA fraction removal** | | | | | **γ-globulin fraction removal** | | | | |
| --- | --- | --- | --- | --- | --- | --- | --- | --- | --- | --- |
|  | **n1** | **n2** | **n3** | **Mean** | **SD** | **n1** | **n2** | **n3** | **Mean** | **SD** |
| 0.0 | 0.0181 | 0.0613 | 0.0166 | 0.0320 | 0.0254 | 0.0680 | 0.0629 | 0.0607 | 0.0639 | 0.0037 |
| 0.1 | 0.1078 | 0.0425 | 0.1034 | 0.0846 | 0.0365 | 0.0428 | 0.0786 | 0.0957 | 0.0724 | 0.0270 |
| 0.2 | 0.2071 | 0.1580 | 0.1934 | 0.1862 | 0.0253 | 0.0719 | 0.1716 | 0.1455 | 0.1297 | 0.0517 |
| 0.3 | 0.3934 | 0.4013 | 0.3961 | 0.3969 | 0.0040 | 0.2647 | 0.4049 | 0.4202 | 0.3633 | 0.0857 |
| 0.4 | 0.6998 | 0.6491 | 0.6796 | 0.6762 | 0.0255 | 0.4508 | 0.4906 | 0.5091 | 0.4835 | 0.0298 |
| 0.5 | 0.9298 | 0.9183 | 0.9044 | 0.9175 | 0.0127 | 0.5472 | 0.5669 | 0.5250 | 0.5464 | 0.0210 |
| 0.6 | 0.9924 | 0.9865 | 0.9944 | 0.9911 | 0.0041 | 0.6010 | 0.6027 | 0.5513 | 0.5850 | 0.0292 |
| 0.7 | 0.9957 | 0.9957 | 0.9961 | 0.9958 | 0.0002 | 0.6363 | 0.5899 | 0.5825 | 0.6029 | 0.0292 |
| 0.8 | 0.9635 | 0.9967 | 0.9957 | 0.9853 | 0.0189 | 0.6552 | 0.6179 | 0.6166 | 0.6299 | 0.0219 |
| 0.9 | 0.9913 | 0.9968 | 0.9966 | 0.9949 | 0.0031 | 0.7140 | 0.5993 | 0.6090 | 0.6408 | 0.0636 |
| 1.0 | 0.9978 | 0.9970 | 0.9968 | 0.9972 | 0.0005 | 0.6925 | 0.6439 | 0.6790 | 0.6718 | 0.0251 |
| 2.0 | 0.9986 | 0.9988 | 0.9983 | 0.9986 | 0.0003 | 0.7640 | 0.6838 | 0.7441 | 0.7306 | 0.0418 |

Fig. 1E

| **NaC9**  **[%w/v]** | **BSA fraction removal** | | | | | **γ-globulin fraction removal** | | | | |
| --- | --- | --- | --- | --- | --- | --- | --- | --- | --- | --- |
|  | **n1** | **n2** | **n3** | **Mean** | **SD** | **n1** | **n2** | **n3** | **Mean** | **SD** |
| 0.0 | 0.0551 | 0.0424 | 0.0304 | 0.0426 | 0.0124 | 0.0761 | 0.0737 | 0.0137 | 0.0545 | 0.0354 |
| 0.1 | 0.1539 | 0.1637 | 0.1784 | 0.1653 | 0.0123 | 0.2184 | 0.2160 | 0.1834 | 0.2059 | 0.0196 |
| 0.2 | 0.4225 | 0.4042 | 0.3447 | 0.3905 | 0.0407 | 0.2740 | 0.3329 | 0.3002 | 0.3024 | 0.0295 |
| 0.3 | 0.6724 | 0.6190 | 0.6229 | 0.6381 | 0.0298 | 0.2850 | 0.3743 | 0.2918 | 0.3170 | 0.0497 |
| 0.4 | 0.8717 | 0.7845 | 0.7856 | 0.8139 | 0.0500 | 0.4056 | 0.3842 | 0.3601 | 0.3833 | 0.0228 |
| 0.5 | 0.9674 | 0.9560 | 0.9519 | 0.9584 | 0.0080 | 0.4366 | 0.3551 | 0.3312 | 0.3743 | 0.0553 |
| 0.6 | 0.9906 | 0.9926 | 0.9908 | 0.9913 | 0.0011 | 0.4449 | 0.4810 | 0.4257 | 0.4505 | 0.0281 |
| 0.7 | 0.9975 | 0.9955 | 0.9960 | 0.9963 | 0.0010 | 0.5266 | 0.4525 | 0.4015 | 0.4602 | 0.0629 |
| 0.8 | 0.9985 | 0.9957 | 0.9968 | 0.9970 | 0.0014 | 0.4670 | 0.4413 | 0.4730 | 0.4604 | 0.0168 |
| 0.9 | 0.9986 | 0.9965 | 0.9975 | 0.9975 | 0.0011 | 0.4784 | 0.4925 | 0.5851 | 0.5187 | 0.0580 |
| 1.0 | 0.9989 | 0.9967 | 0.9974 | 0.9977 | 0.0011 | 0.5613 | 0.5332 | 0.5096 | 0.5347 | 0.0259 |
| 2.0 | 0.9989 | 0.9977 | 0.9980 | 0.9982 | 0.0006 | 0.7036 | 0.7231 | 0.6655 | 0.6974 | 0.0293 |

Fig. 1F

| **NaC10**  **[%w/v]** | **BSA fraction removal** | | | | | **γ-globulin fraction removal** | | | | |
| --- | --- | --- | --- | --- | --- | --- | --- | --- | --- | --- |
|  | **n1** | **n2** | **n3** | **Mean** | **SD** | **n1** | **n2** | **n3** | **Mean** | **SD** |
| 0.0 | 0.0648 | 0.0791 | 0.0087 | 0.0509 | 0.0372 | 0.0970 | 0.1215 | 0.0519 | 0.0901 | 0.0353 |
| 0.1 | 0.2406 | 0.1546 | 0.1570 | 0.1841 | 0.0490 | 0.2303 | 0.2019 | 0.1850 | 0.2057 | 0.0229 |
| 0.2 | 0.4466 | 0.3425 | 0.3771 | 0.3887 | 0.0530 | 0.2881 | 0.2691 | 0.2325 | 0.2632 | 0.0283 |
| 0.3 | 0.6415 | 0.6060 | 0.6183 | 0.6219 | 0.0180 | 0.3757 | 0.3229 | 0.2491 | 0.3159 | 0.0636 |
| 0.4 | 0.8003 | 0.7437 | 0.7613 | 0.7684 | 0.0290 | 0.3826 | 0.2808 | 0.3895 | 0.3510 | 0.0609 |
| 0.5 | 0.8595 | 0.8545 | 0.8544 | 0.8561 | 0.0029 | 0.3891 | 0.3381 | 0.4407 | 0.3893 | 0.0513 |
| 0.6 | 0.9194 | 0.8777 | 0.9000 | 0.8990 | 0.0209 | 0.3757 | 0.4539 | 0.3495 | 0.3930 | 0.0543 |
| 0.7 | 0.9359 | 0.9314 | 0.9097 | 0.9257 | 0.0140 | 0.3525 | 0.4446 | 0.3430 | 0.3800 | 0.0561 |
| 0.8 | 0.9174 | 0.9240 | 0.8414 | 0.8943 | 0.0459 | 0.4347 | 0.4544 | 0.3954 | 0.4282 | 0.0300 |
| 0.9 | 0.9521 | 0.9545 | 0.9159 | 0.9408 | 0.0216 | 0.4886 | 0.4965 | 0.4435 | 0.4762 | 0.0286 |
| 1.0 | 0.9414 | 0.9533 | 0.9594 | 0.9514 | 0.0092 | 0.5089 | 0.5291 | 0.4494 | 0.4958 | 0.0414 |
| 2.0 | 0.9378 | 0.9105 | 0.8332 | 0.8938 | 0.0543 | 0.7441 | 0.7441 | 0.7194 | 0.7359 | 0.0143 |

Fig. 4

| **γ-globulin sample/ Precipitant** | **Fluorescence intensity [au.] @475 nm.** | | | | |
| --- | --- | --- | --- | --- | --- |
|  | **n1** | **n2** | **n3** | **Mean** | **SD** |
| Positive control* | 22.751 | 20.850 | 19.875 | 21.159 | 1.463 |
| Untreated | 3.037 | 3.467 | 3.168 | 3.224 | 0.220 |
| C8 [% v/v] | | | | | |
| 0.5 | 4.004 | 4.586 | 3.820 | 4.137 | 0.400 |
| 1 | 3.927 | 4.031 | 3.754 | 3.904 | 0.140 |
| C9 [% v/v] | | | | | |
| 0.5 | 3.556 | 3.533 | 3.523 | 3.537 | 0.017 |
| 1 | 3.263 | 3.612 | 3.713 | 3.529 | 0.236 |
| C10 [%v/v] | | | | | |
| 0.5 | 3.633 | 3.332 | 3.655 | 3.540 | 0.180 |
| 1 | 3.331 | 3.995 | 3.622 | 3.649 | 0.333 |
| NaC8 [%w/v] | | | | | |
| 0.5 | 5.389 | 4.106 | 3.851 | 4.449 | 0.824 |
| 1 | 4.312 | 3.394 | 3.849 | 3.852 | 0.459 |
| NaC9 [%w/v] | | | | | |
| 0.5 | 3.426 | 3.341 | 3.699 | 3.489 | 0.187 |
| 1 | 4.055 | 3.906 | 4.285 | 4.082 | 0.191 |
| NaC10 [%w/v] | | | | | |
| 0.5 | 3.221 | 3.551 | 3.455 | 3.409 | 0.170 |
| 1 | 3.829 | 3.519 | 3.521 | 3.623 | 0.178 |

*Positive control = Heat treated γ-globulin at 95°C for 5 minutes

Fig. 7A

| **Sample** | **Relative OD [%] @450 nm.** | | | | |
| --- | --- | --- | --- | --- | --- |
|  | **n1** | **n2** | **n3** | **Mean** | **SE** |
| Crude plasma | 63.87 | 64.83 | 62.58 | 63.76 | 1.13 |
| Ref. C8 | 61.67 | 56.51 | 59.48 | 59.22 | 2.59 |
| 1%C8 | 89.48 | 85.42 | 83.58 | 86.16 | 3.02 |
| 1%C9 | 72.99 | 75.75 | 75.31 | 74.68 | 1.48 |
| 1%C10 | 83.88 | 74.33 | 79.35 | 79.19 | 4.78 |
| 1%NaC8 | 59.46 | 64.14 | 69.54 | 64.38 | 5.04 |
| 1%NaC9 | 55.43 | 56.81 | 56.72 | 56.32 | 0.77 |
| 1%NaC10 | 54.25 | 59.48 | 60.10 | 57.94 | 3.21 |

Fig. 7B

| **Sample** | **Relative OD [%] @450 nm.** | | | | |
| --- | --- | --- | --- | --- | --- |
|  | **n1** | **n2** | **n3** | **Mean** | **SE** |
| Crude plasma | 63.87 | 64.83 | 62.58 | 63.76 | 1.13 |
| Ref. C8 | 61.67 | 56.51 | 59.48 | 59.22 | 2.59 |
| 2%C8 | 61.39 | 60.57 | 58.27 | 60.08 | 1.62 |
| 2%C9 | 67.40 | 73.29 | 69.12 | 69.94 | 3.03 |
| 2%C10 | 72.47 | 67.29 | 65.60 | 68.45 | 3.58 |
| 2%NaC8 | 49.27 | 50.91 | 44.33 | 48.17 | 3.43 |
| 2%NaC9 | 44.22 | 41.38 | 43.78 | 43.13 | 1.53 |
| 2%NaC10 | 43.78 | 42.05 | 40.73 | 42.19 | 1.53 |
